# Minimization of Immunosuppressive Therapy Is Associated with Improved Survival of Liver Transplant Patients with Recurrent Hepatocellular Carcinoma

**DOI:** 10.3390/cancers13071617

**Published:** 2021-03-31

**Authors:** Ramin Raul Ossami Saidy, Maximilian Paul Postel, Michael Johannes Pflüger, Wenzel Schoening, Robert Öllinger, Safak Gül-Klein, Moritz Schmelzle, Frank Tacke, Johann Pratschke, Dennis Eurich

**Affiliations:** 1 Department of Surgery, Campus Virchow-Klinikum and Campus Charité Mitte, Charité University Medicine Berlin, Augustenburger Platz 1, 13353 Berlin, Germany; ramin-raul.ossami-saidy@charite.de (R.R.O.S.); maximilian.postel@charite.de (M.P.P.); michael.pflueger@charite.de (M.J.P.); wenzel.schoening@charite.de (W.S.); robert.oellinger@charite.de (R.Ö.); safak.guel@charite.de (S.G.-K.); moritz.schmelzle@charite.de (M.S.); johann.pratschke@charite.de (J.P.); 2 Department of Hepatology and Gastroenterology, Charité University Medicine Berlin, 10117 Berlin, Germany; frank.tacke@charite.de

**Keywords:** liver transplantation, recurrent hepatocellular carcinoma, immunosuppression, long-term follow-up, surgical tumor resection

## Abstract

**Simple Summary:**

Liver transplantation is a curative treatment option for a subset of patients with hepatocellular carcinoma (HCC). However, about twenty percent of patients develop recurrence in the graft or at extrahepatic sites, which is associated with limited therapeutic options and poor survival. To date, management of the immunosuppressive regimen after recurrence and its impact on survival are unknown. In this retrospective study, we analyzed a cohort of liver recipients with HCC recurrence. Our findings indicate that reduction of immunosuppressive therapy after diagnosis of recurrence has a beneficial impact on patient survival. Therefore, we propose further investigation into the management of immunosuppressive therapy following recurrence.

**Abstract:**

Introduction: Recurrence of hepatocellular carcinoma (rHCC) after liver transplantation (LT) is associated with limited survival. Therefore, identification of factors that prolong survival in these patients is of great interest. Surgical resection, radiotherapy, and transarterial chemoembolization (TACE) are established interventions to improve outcomes in these patients; however, the impact of immunosuppression is unknown. Methods: All patients diagnosed with rHCC in the follow-up after LT were identified from a database of liver recipients transplanted between 1988 and 2019 at our institution (Charité Universitätsmedizin Berlin, Germany). Based on the immunosuppressive regimen following diagnosis of rHCC and the oncological treatment approach, survival analysis was performed. Results: Among 484 patients transplanted for HCC, 112 (23.1%) developed rHCC in the follow-up. Recurrent HCC was diagnosed at a median interval of 16.0 months (range 1.0–203.0), with the majority presenting early after transplantation (63.0%, <2 years). Median survival after rHCC diagnosis was 10.6 months (0.3–228.7). Reduction of immunosuppression was associated with improved survival, particularly in patients with palliative treatment (8.4 versus 3.0 months). In addition, greater reduction of immunosuppression seemed to be associated with greater prolongation of survival. Graft rejection after reduction was uncommon (*n* = 7, 6.8%) and did not result in any graft loss. Patients that underwent surgical resection showed improved survival rates (median 19.5 vs. 8.7 months). Conclusion: Reduction of immunosuppressive therapy after rHCC diagnosis is associated with prolonged survival in LT patients. Therefore, reduction of immunosuppression should be an early intervention following diagnosis. In addition, surgical resection should be attempted, if technically feasible and oncologically meaningful.

## 1. Introduction

Increasing incidence rates of hepatocellular carcinoma (HCC) require efficient diagnostic and therapeutical approaches. Main etiologies for HCC remain viral hepatitis, alcohol abuse, and non-alcoholic fatty liver disease, resulting in cirrhosis and eventually HCC. If the tumor is localized, liver transplantation (LT) offers a chance for cure.

Recurrence of HCC (rHCC) following liver transplantation is a major complication and affects about 20% of recipients, indicating failure of the primary treatment approach [1]. Previous research highlighted alpha-fetoprotein (AFP) levels, low histologic cell differentiation, microvascular invasion, and short time on the waitlist as potential risk factors for recurrence [2,3]. In addition, immunosuppressive agents have been thoroughly studied to elucidate their role in the development of rHCC [4].

Calcineurin inhibitors (CNIs) and mammalian target of rapamycin inhibitors (mTORIs) are most frequently used as standard immunosuppression (IS). Beyond well-known side effects of CNIs, such as kidney dysfunction and metabolic disorders, the impact on tumor development and rHCC is controversial [5]. The mTORI were thought to become the flagship in IS management because of their anti-proliferative and anti-angiogenetic properties, which may be favorable to prevent recurrence [4,6,7]. In fact, there is some evidence about the advantages of mTORI use in patients with low-risk profiles, young age (<60 years), and monotherapy regarding recurrence free survival (RFS) over 5 years; but there is still some uncertainty about which IS regimen is best for prevention of rHCC after LT [8].

Prognosis of rHCC is poor with a mean survival of only one year following diagnosis, and individual treatment schedules are needed that consider tumor stage, functional capacity of the graft, and general performance status [2]. Early stages may be treated by surgical resection with or without chemoembolization, whereas advanced stages with disseminated tumor manifestation can be treated by tyrosine kinase inhibitors, such as sorafenib or lenvatinib, or best supportive care (BSC) [2]. Checkpoint inhibitors such as nivolumab, a PD-1-blocker and immunomodulator, may lead to graft rejection by decreasing the activity of regulatory T-cells [9].

Intriguingly, the impact of immunosuppression on survival in patients with rHCC is yet unknown. Substance class changes to mTORIs and dose reductions of IS are discussed, but data on this topic is very limited and needs further scientific evidence. Thus, no recommendations exist in current guidelines [2,9]. The aim of this study was to investigate factors associated with improved survival of liver transplant recipients after diagnosis of rHCC, with a particular emphasis on the impact of IS.

## 2. Patients and Methods

All patients who underwent LT for HCC at our institution (Department of Surgery, Charité Universitätsmedizin Berlin) between 1988 and 2019 were considered for the analysis. Diagnosis of HCC was histopathologically confirmed, and patients with cholangiocellular carcinoma (CCC) or mixed-carcinoma were excluded from analysis.

All patients were followed-up periodically at our outpatient center both clinically (including serological tests) and radiographically. Follow-up intervals were based on the time after transplantation and ranged between 2×/week to every twelve weeks. Ultrasound-guided, transcostal needle core biopsies of the graft were routinely performed according to our standard protocol at 1, 3, 5, 7, 10, and 13 years and on a case-by-case basis thereafter. Biopsy specimens were assessed by trained pathologists, and grade of fibrosis was categorized as follows: 0—absent; 1—mild portal fibrosis; 2—moderate with few incomplete portal septa; 3—numerous portal septa without architectural disturbances; and 4—cirrhosis [10].

Recipients transplanted for HCC were followed-up with alpha-fetoprotein (AFP) levels taken every 6 months. This was combined with abdominal ultrasound in a life-long surveillance concept. In concordance with recent guidelines, cross-sectional imaging (i.e., CT, MRI) was performed every 6 months for the first 2 years and in cases of suspected rHCC. Diagnosis of rHCC was made by experienced radiologists based on established radiographical criteria in CT or MRI (e.g., strong enhancement in hepatic arterial phase, wash-out in portal venous and/or equilibrium phase, hypervascular lesions) [11]. Imaging-guided biopsies were taken to confirm the diagnosis if radiographic assessment alone was inconclusive and surgical resection was not feasible. Diagnosis of rHCC and therapeutic options were discussed in a multidisciplinary tumor board.

Early-onset rHCC and late-onset rHCC were defined as less or more than 2 years following LT, respectively. Histological grading of HCC was determined by pathological findings of the initial HCC using the Edmondson–Steiner classification: 1—minimal nuclear irregularity, 2—greater nuclear irregularity and prominent nucleoli, 3—increased nuclear polymorphism and tumor giant cells, and 4—poorly differentiated with hyperchromatism and anaplasia [12]. The extent of rHCC was dichotomized into single-organ manifestation (SOA) or disseminated recurrence (MOA). Surgical resection, radiotherapy, chemotherapy, targeted therapy, or BSC were recorded. All interventional methods or local ablation procedures performed under radiographical guidance were categorized as “radiotherapy” for this study. This included radiation therapy, transarterial chemoembolization (TACE), radio frequency ablation (RFA), microwave ablation (MWA), and alcohol instillation. Patients with recurrence as mixed hepatocellular and cholangiocellular carcinoma were excluded.

Recurrence of hepatitis C-virus (HCV)- or hepatitis B-virus (HBV)-infection was diagnosed with new increases of HCV-RNA or HBV-DNA copies in polymerase chain reaction (PCR), respectively.

To account for potential changes in IS regimen before and after diagnosis of rHCC, we grouped IS of the patients in two categories for our analysis: (i) maintaining immunosuppression, or (ii) new restrictive immunosuppressive management (RIM). RIM was defined as significant dose reduction or complete discontinuation of IS after diagnosis of rHCC. If mTOR therapy was initiated without reduction of prior IS, the regimen was classified as (i); if concomitant reduction of IS was documented, patients were grouped in (ii). Follow-up data were acquired by in-hospital data and reports from outside institutions (including primary care physicians, local gastroenterologists, and oncologists). Data on clinical, laboratory, and histological parameters were extracted from a prospectively maintained database and evaluated retrospectively, depending on the study questions related to rHCC.

For multivariate analysis and consideration of confounding variables, the approach was based on clinical/biological relevant putative confounding parameters with potential relevant additional damage to the liver (e.g., hepatitis, diabetes, obesity) or patients’ course (e.g., age).

Statistical analysis was performed using SPSS Statistics Version 25.0 (IBM Co., Armonk, NY, USA), and an exploratory study design was used. Cross-tables were used for testing of differences in nominal variables and *t*-test for normally distributed continuous variables. Mann–Whitney *U*-test or Kruskal–Wallis test were performed for testing of non-normally distributed values. Univariate analysis and Kaplan–Meier analysis were performed to compare and illustrate survival differences and both Breslow- and log rank-test were calculated to evaluate the short- and long-term effect, respectively.

Both multivariate and univariate Cox-regression-models were used to evaluate effect strength, with a hazard ratio (HR) of less than one indicating survival benefit. A *p*-value of <0.05 was considered significant.

## 3. Results

In total, 484 patients had undergone liver transplantation for histologically proven HCC post explant between 1988–2019. Of these, 112 patients (23.1%) developed rHCC. The majority of this recurrence cohort was male (*n* = 100; 89.3%), reflecting the male predominance in the cohort of all patients that underwent LT for HCC in this period (394/484; 81.4%). Main etiologies of primary HCC were HCV-, HBV-, and alcohol-associated and cryptogenic cirrhosis. Three patients (2.7%) with loss of graft function underwent re-transplantation prior to the diagnosis of rHCC. Follow-up data for this study were analyzed until April 2020, and only nine patients (8.0%) were alive at this point. Table 1 provides further details of the patient cohort.

**Figure 1:**
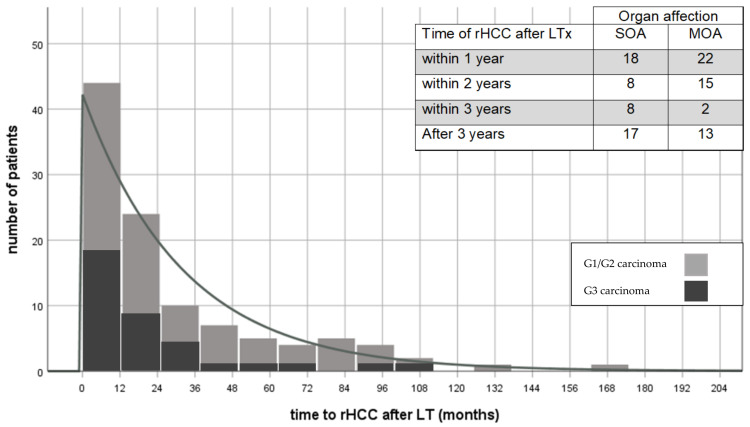
Recurrence of HCC after liver transplantation and manifestation at time of diagnosis. rHCC—recurrent hepatocellular carcinoma; LT—liver transplantation; SOA—single-organ affection; MOA—multi-organ affection.

**Figure 2:**
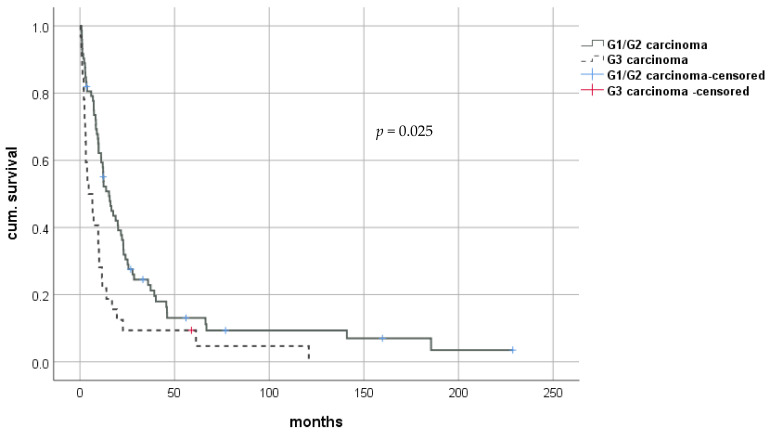
Survival after diagnosis of rHCC after LT for HCC. rHCC—recurrent hepatocellular carcinoma; LT—liver transplantation.

Median time to rHCC was 16.0 months (1.0–203.0). Almost two-third of our study population developed rHCC as early-onset rHCC (<2 years; 63.0%). However, rHCC occurred in the third year after LT in 10 patients (9.7%), between 4 and 10 years in 27 patients (26.2%), and beyond 10 years in 3 patients (2.9%; at 11, 14, and 16-years, respectively) (Figure 1). Median survival after diagnosis of rHCC was 10.6 months (0.3–228.7; Q1–Q3: 3.3–22.9). Patients with early recurrence (<2 years) had a significantly shorter median survival after rHCC diagnosis than patients with late onset of rHCC (9.5 months (0.8–66.3; interquartile range (IQR) 15.3) versus 15.5 months (0.3–228.7; IQR 37.7), respectively; *p* < 0.001). Kaplan–Meier analysis revealed survival benefits for patients in the group of late-onset rHCC (log-rank *p* < 0.001; Breslow *p* < 0.001). Of the 72 patients with G1/G2 carcinoma, 42 (58.3%) had early-onset of recurrence. In contrast, 23 of the 32 patients with G3 carcinoma (71.9%) had early-onset of recurrence. This trend continued, with 48 patients (66.7%) with G1/G2 carcinoma and 27 patients (84.4%) with G3 carcinoma being diagnosed with recurrence at 36 months post transplantation. Histological grading showed significant differences in overall survival after liver transplantation with medians of 37.5 (2.1–288.3; IQR 55.7) months for G1/G2 tumors compared to 23.5 (5.1–209.3; IQR 36.7) months for G3 tumors (log-rank *p* = 0.025; Breslow *p* = 0.004). The survival benefit for patients with lower-grade tumors was still present when restricting the analysis for survival after diagnosis of rHCC with median survival of 12.4 (0.8–228.7; IQR 20.4) months for G1/G2 tumors and 5.6 (0.3–120.9; IQR 9.4) months for G3 tumors (log-rank *p* = 0.002; Breslow *p* < 0.001) (Figure 2).

Recurrence of the disease that led to development of HCC occurred in 41 (42.7%) recipients and was HCV in 33, HBV in 6, and alcohol abuse in 2 cases. All six patients with recurrent HBV-infection were treated with nucleos(t)id-analogs, predominantly with lamivudine. Of the 33 patients with HCV-recurrence, interferon-based antiviral therapy was performed in 10 patients (30.3%), and in 23 patients (69.7%) no therapy was initiated or was prematurely stopped due to side-effects. Biopsies of the graft were available in 21 patients (53.8%) and fibrosis stage 1 was seen in 4 patients (10.3%), stage 2 in 13 patients (33.3%), and in 2 patients (5.1%) numerous portal septa–stage 3–were found. No manifest cirrhosis was documented. No significant statistical difference was found in survival analysis between group of recurrence of the underlying disease and those without evidence of recurrent disease (*n* = 71) after the diagnosis of rHCC, neither when comparing the subgroups (log-rank *p* = 0.9; Breslow *p* = 0.87) nor overall (log-rank *p* = 0.67; Breslow *p* = 1.0).

Nine patients (8.0%) were lost to follow-up, resulting in 103 cases in which sufficient data were available for analysis of treatment of rHCC. Most frequently involved organs were the liver graft (*n* = 48/42.9%), lungs (*n* = 48/42.9%), bone (*n* = 35/31.3%), and other intraabdominal organs (including adrenal gland; *n* = 29/25.9%). Single-organ affection (SOA) was recorded in 51 cases (49.5%) and multiple-organ affection (MOA) in 52 patients (50.5%). In Kaplan Meier analysis, long-term survival was significant longer in case of SOA (log-rank *p* = 0.007). There was no significant difference between manifestation of rHCC as SOA and MOA in a time dependent manner at time of diagnosis (*p* = 0.084) (Figure 1).

**Figure 3:**
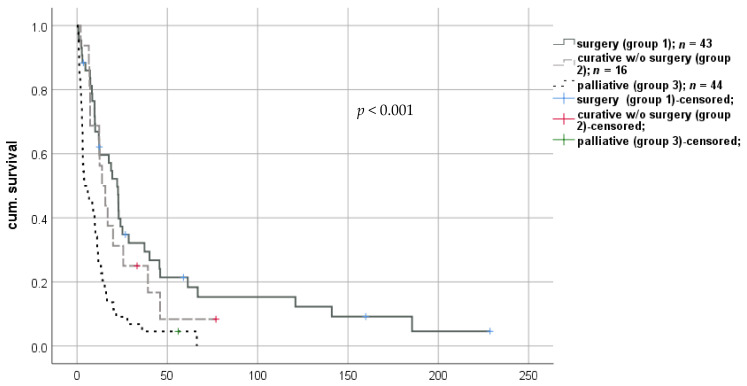
Impact of therapeutical strategy on survival after diagnosis of rHCC after liver transplantation. rHCC—recurrent hepatocellular carcinoma.

Therapy strategies after diagnosis of rHCC were surgery, radiotherapy, systemic anti-neoplastic therapies, including traditional chemotherapy and/or pharmacological targeted therapies, and BSC without additional interventions. Therefore, three main groups were formed: A curative regimen was initiated in 59 patients (57.3%), of which 43 were treated surgically with curative intent (group 1) and 16 patients (15.5%) with a local-ablative, curatively intended treatment approach (group 2). In contrast, 44 patients (42.7%) received palliative treatment after diagnosis of rHCC (e.g., palliative chemotherapy/targeted therapy or BSC) due to advanced disease or reduced physical status (group 3; Figure 3).

The palliative group had a significantly shorter median survival compared to all other treatment regimens (9.6 months (0.3–66.2; IQR 10.4) vs. 13.6 months (0.8–159.8; IQR 29.1), respectively; log-rank *p* < 0.001; Breslow *p* < 0.001).

**Figure 4:**
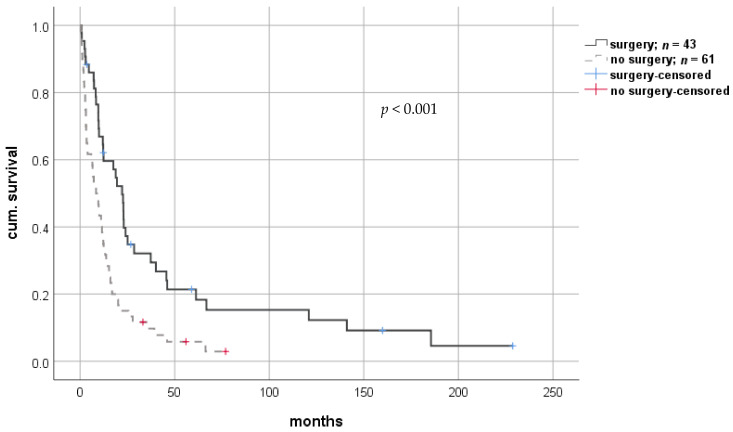
Comparison of overall survival of patients with or without surgical tumor resection after diagnosis of rHCC. rHCC—recurrent hepatocellular carcinoma.

Twenty-seven patients (26.2%) underwent (interventional) radiotherapy, including TACE. This group showed a significant advantage in short-term survival analysis compared to all others (median 17.0 months (2.0–70.9; IQR 15.5) vs. 9.7 months (0.3–228.7; IQR 20.0), respectively; log-rank *p* = 0.364; Breslow *p* = 0.042). Of the 43 patients that underwent surgical resection, 21 patients (48.8%) were diagnosed with SOA, and 22 patients (51.2%) were diagnosed with MOA. These manifestations of rHCC did not differ between patients undergoing and not undergoing surgical tumor resection (*p* = 1.0). Kaplan–Meier analysis showed significantly improved short- and long-term survival for patients that underwent surgery compared to those that did not (median 19.5 months (0.8–228.7; IQR 31.7) vs. 8.7 months (0.3–76.9; IQR 13.1), respectively; log-rank *p* < 0.001; Breslow *p* < 0.001) (Figure 4). Similarly, patients who underwent surgical therapy had a significant survival benefit compared to those without the surgical approach, with a hazard ratio of 0.468 in Cox-regression analysis (CI: 0.303–0.722; *p* = 0.001). When adjusted for potential confounders such as age or comorbidities in multivariate analysis, this statistical finding was confirmed (Table 2).

A subgroup analysis comparing the survival between curative therapy regimens (i.e., surgery only, radiotherapy only, multimodal combination of radiotherapy with surgery and/or systemic therapy) did not yield statistically significant differences in survival.

Prior to diagnosis of rHCC, 92 patients (89.3%) were exposed to CNIs, and in 77 cases (74.8%), CNIs remained part of IS after rHCC diagnosis. In 85 patients (82.5%), tacrolimus was administered with a mean dosage of 2.8 mg per day and median trough level of 5.9 ng/mL (Q1–Q3: 1.9–5.8 ng/mL). In 41 cases (39.0%), mycophenolate mofetil (MMF) was part of the immunosuppressive regimen with a mean dosage of 1.4 g per day, and 21 patients (20.4%) were given mTORI with a mean dosage 2.6 g per day. Prednisolone was given to 16 patients (15.5%) with a mean dosage of 8.5 mg per day. Only 7 patients (6.8%) received cyclosporine A (CYA) with a mean dosage of 195.7 mg per day and median trough level of 185 (73–287; Q1–Q3: 129.0–270.0) µg/L.

After diagnosis of rHCC, the number of patients who received mTORI increased from 21 (20.4%) to 37 (35.9%). No statistically significant difference was found in survival for patients with preexisting mTORI therapy compared to all others (log-rank *p* = 0.149; Breslow *p* = 0.288). A similar observation in long-term survival was found in 22 patients (21.4%) that were converted to mTORI after diagnosis of rHCC. Here, however, short-term survival was improved with statistical significance (log-rank *p* = 0.11; Breslow *p* = 0.009).

Overall, modifications of immunosuppressive therapy after diagnosis of rHCC were frequent. A restrictive immunosuppressive management (RIM), i.e., discontinuation or significant dose reduction after diagnosis of rHCC, was conducted in 69 patients (67.0%). Most commonly, tacrolimus was reduced in 56 patients (54.4%), from a mean dosage of 3 mg per day and trough level of 5.9 ng/mL at the time of rHCC diagnosis to 1.5 mg per day and trough level of 3.2 ng/mL after diagnosis of rHCC. Analysis for paired testing found this reduction to be statistically significant (*p* < 0.001). Similarly, reduction of MMF dosage was performed in 27 patients (26.2%) from 1.4 g per day to 0.6 g per day and found to be statistically significant (*p* < 0.001). Reduction of prednisolone from 7.7 mg per day to 2 mg per day on average (*p* = 0.07) and CYA from 150 mg per day to 50 mg per day both did not reach statistical significance (*p* = 0.12). This was likely due to the low number of patients under these specific regimens.

**Figure 5:**
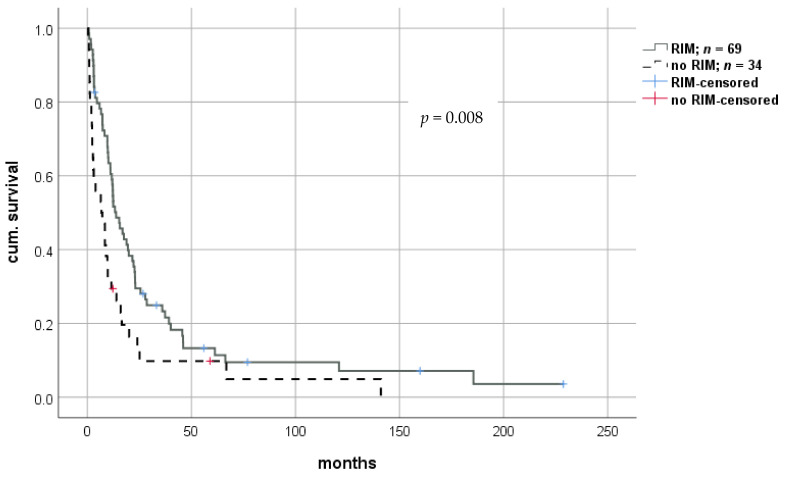
Impact of a restrictive immune suppressive management (RIM) after the diagnosis of rHCC after liver transplantation. rHCC—recurrent hepatocellular carcinoma.

**Figure 6:**
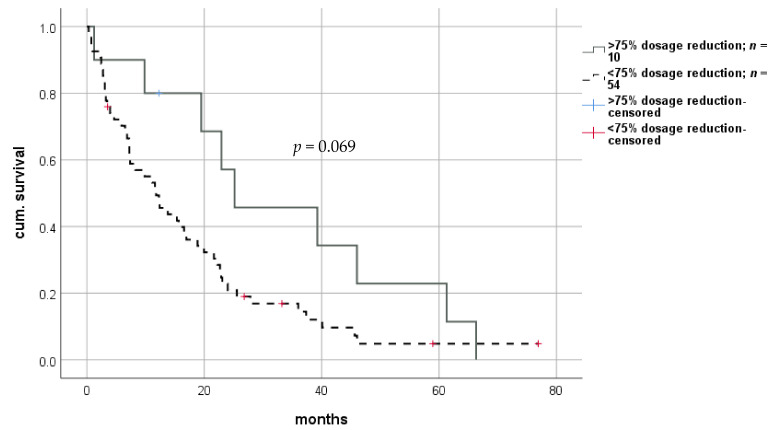
Impact of dosage reduction on survival after diagnosis of rHCC after liver transplantation. rHCC—recurrent hepatocellular carcinoma.

Both short- and long-term survival were significantly improved using the RIM approach, with a median survival of 13.2 months (0.8–228.7; IQR 21.3) in patients with RIM versus 7.0 months (0.3–141.0; IQR 12.4) in patients without RIM (log-rank *p* = 0.008; Breslow *p* = 0.001). Thus, we observed a mean prolongation of survival of 5.5 months in patients whose immunosuppression levels were significantly reduced (Figure 5). Cox-regression revealed a hazard ratio of 0.562 (CI: 0.365–0.722; *p* = 0.009). Again, this effect remained significant in multivariate analysis (Table 2). Survival benefit seemed to be highest in patients undergoing extensive reduction of IS, indicating a dose-dependent effect. However, statistical analysis did not reach significance (Figure 6). Graft rejection in patients with RIM occurred in seven cases (6.8%) and was treated with intravenous methylprednisolone and/or moderate increase in immunosuppressive dose. Importantly, no graft loss occurred.

In a subgroup analysis of patients with a curative therapy approach only, RIM did not show a statistically significant impact on survival using Kaplan–Meier analysis (log-rank *p* = 0.413; Breslow *p* = 0.105). A total of 45 patients received both RIM and curative therapy and had a median survival of 19.5 months (0.8–228.7; IQR 28.4). The group of 14 patients with curative therapy only had a median survival of 7.8 months (1.0–141.0; IQR 23.9). In addition, no significant differences were found when patients with a curative regimen were split further into surgery or local-ablative therapy.

A statistically prolonged survival was found in the 44 patients of the palliative group when RIM was applied (log-rank *p* = 0.020; Breslow *p* = 0.027). In 24 patients, an immunosuppressive regimen was reduced, and in 20 remained unchanged with median survival intervals of 8.4 (1.0–66.3; IQR 12.3) months and 3.0 (0.3–20.1; IQR 19.8) months, respectively.

Overall, 33 patients (32.0%) received a curative oncological regimen with surgical tumor resection and additional intervention (e.g., TACE, targeted therapy) together with RIM. The patients with this treatment combination showed significantly improved survival compared to all other patients using Kaplan–Meier survival analysis (log-rank *p* = 0.010; Breslow *p* = 0.003) with median survival of 19.5 months (0.8–228.7; IQR 29.1) and 12.4 months (1.0–141.0; IQR 14.7), respectively.

In an additional subgroup analysis, we identified 17 patients (16.5%) who received surgical tumor resection without additional radiotherapy or chemo/targeted therapy as oncological treatment plus RIM. Univariate analysis also showed statistically significant longer survival in this group (median 22.7 months (0.8–228.7; IQR 71.3) vs. 11.2 months (0.3–141.0; IQR 18.6); log-rank *p* = 0.009; Breslow *p* = 0.041).

## 4. Discussion

Recurrent HCC is a life-threatening oncological complication after LT, which if not diagnosed at an early stage, has limited curative options with median survival of less than 12 months, consistent with our findings [13,14]. While previous studies targeted risk factors for the occurrence of HCC after LT, the present study aims to identify factors that impact patient survival when diagnosis of recurrent HCC (rHCC) is already established. The findings of this retrospective analysis of 103 recipients with rHCC collected at a single-center over 30 years indicate that surgical therapy and individualized reduction of immunosuppressive therapy after rHCC diagnosis significantly improve patient survival. Although these associations may sound intuitive, to the best of our knowledge there are no data on this aspect in literature.

The main results of the present analysis that were associated with a longer survival of LT recipients after diagnosis of rHCC refer to an early diagnosis of recurrence, the technical feasibility of surgical therapy, and the positive effect of reduced immunosuppression. Similarly, radiotherapy has been shown to be effective, and we observed a beneficial effect on short-term survival in our cohort comparable to other studies [15,16].

Surgical resection of rHCC after LT has been reported to be the only curative strategy and thus should be the first-line approach if the patient is fit to undergo surgery [9,17,18,19]. Intriguingly, in the present study, the cohort of patients undergoing surgery was not confounded by the extent of rHCC, with an even distribution of single- and multi-organ affection at time of diagnosis. Survival benefit of surgical resection has been demonstrated in the current study, and safety has been shown previously by our group and others [17,20]. Therefore, recipients should be followed-up in a risk-adapted fashion to detect recurrence at a time when surgical resection is still feasible. Particularly, patients with high-grade HCCs (G3) should be closely monitored. In Germany, current guidelines suggest radiographic and serological (i.e., AFP levels) HCC surveillance for 2 years following LT [21]. Based on our experience with almost forty percent of all patients suffering from rHCC recurring within 2 years (40/103; 39%), recurrence risk remains significantly elevated even after the guideline-recommended interval of 2 years. Of note, a quarter of patients experienced recurrence between 3 and 10 years (27/103; 26%). Given that the patients were closely followed-up in our outpatient center with a particular focus on the recurrence risk, and given that diagnostic resources for further work-up were readily available, we think it is unlikely that these findings reflect delayed diagnosis. Therefore, we advocate for sustained HCC surveillance well beyond 2 years [22].

Optimal immunosuppressive regimen for recipients transplanted for HCC lacks definitive evidence, although recommendations for treatment of this population with a combination of CNI and mTORI are strong [9,16,23]. The anti-proliferative effect of mTORIs (e.g., everolimus) is thought to prevent recurrence of HCC, thus making these substances superior candidates for life-long immune suppression after LT. Several studies show at least positive effects of mTORIs in rHCC occurrence or organ toxicity, although the latter is thought to be mainly due to concomitant reduction of CNIs [24,25,26,27]. A randomized controlled trial comparing sirolimus with mTORI-free immunosuppression showed no benefit regarding HCC recurrence in the entire study population, whereas an exploratory analysis found improved survival when mTORI were administered for more than three months after LT [8,23]. In our study, we only observed improvement in short-time survival for patients receiving mTORI. However, the effect of mTORI in prevention of rHCC was not the main objective in the present analysis.

Immunosuppressive therapy after LT remains highly individualized with choice of the substances, their dosage, preparation, and combinations being based on each recipients’ comorbidities, individual metabolism, and short- and long-term side effect profiles [28,29,30]. However, the backbone of IS in former HCC patients consists of tacrolimus, MMF, and mTORI, while CYA and corticosteroids are generally no longer used in long-term immune suppression.

There is a particular interest in reducing serum drug levels of immunosuppressants to avoid long-term side effects, such as nephrotoxicity, infections, or neoplasms [9,31,32]. In addition, high dosages of immunosuppressants are linked to recurrence [7,33,34]. Drug levels of tacrolimus-based immunosuppression have become significantly lower in the modern era, particularly in patients with stable long-term graft function. In a subset of patients, IS was even successfully discontinued, thus potentially reducing the risk for infections, neoplasms, and metabolic disorders [35].

To date, there is no guideline addressing the management of IS after diagnosis of rHCC or other neoplasms in solid-organ recipients, and its impact remains unclear, despite the known contribution of IS intensity to cancer recurrence [6,9]. The hypothesis that reduction of immunosuppression allows for improved tumor recognition and enhances possible anti-neoplastic features of the immune systems is based on a strong biological plausibility, which is comparable to modern biologicals in a non-transplant setting. Of note, these substances are currently not approved for transplant recipients. In our study, we did not observe any major side effects or organ loss after initiation of an individualized RIM but found a significant survival benefit of 5.5 months as compared to patients, in which IS was not altered after rHCC diagnosis. In addition, a dosage-dependent survival benefit was observed. Given that the overall survival was 10.6 months in the entire cohort, these findings illustrate the leverage changes in immunosuppression have on survival in rHCC. Multivariate Cox-regression was performed to identify one (or more) particularly beneficial therapies among the various options. Unfortunately, effect sizes were low due to small subgroups.

When analyzing subgroups, the effect of RIM in our population was only statistically significant in the group that underwent a palliative regimen. However, since 43% of our cohort received a palliative regimen, this is an important finding that affects a sizable portion of patients with rHCC. Given the limited treatment options in this group and their particular benefit from RIM, dosage reduction of IS seems mandatory in these cases. Moreover, there was a remarkable difference between patients with curative approach and RIM as compared to those with a curative approach without RIM (median 19.5 months versus 7.8 months). Failure to meet statistical significance for this observation was likely due to the small number of patients in both groups.

Based on the study findings and our clinical experience, we established an internal institutional standard operating procedure that combines RIM with close surveillance in an interdisciplinary approach. Further individualization of each patients’ therapy is needed in these cases to improve limited survival. However, proper evaluation of the oncological strategy, particularly the feasibility of surgical resection or interventional therapy, have to be conducted without exception.

Our study is limited by its retrospective approach, origin from a single center, and a three-decade span of varying therapeutical regimens, the latter serving as strength and limitation at the same time. Because of the diversity of immunosuppressive regimens, potential confounders such as physical constitution prior to rHCC cannot be excluded, and secondary endpoints, such as quality of life, were not assessed. Furthermore, no standard regimen concerning reduction existed, and RIM was termed in a retrospective analysis and no threshold or algorithm existed at time of reduction of IS. Thus, a heterogeneity of dosage reduction has to be acknowledged, and appropriate dosage-finding for reduction has to be evaluated in the future.

## 5. Conclusions

The findings of this study indicate that close surveillance, which exceeds the currently recommended span of 2 years, is required for LT recipients that were previously transplanted for HCC. In light of the improved survival associated with surgical resection, early detection is critical to allow for diagnosis of recurrent tumors at a resectable stage. In addition, initiation of RIM after diagnosis of rHCC is associated with prolonged survival, particularly in patients with a palliative situation. Given the limited treatment options, reduction of IS seems to be a practical and cost-effective step to induce a survival benefit and can be done safely. To guide physicians in modification of IS after rHCC diagnosis, we propose the possibility to reduce immunosuppression up to discontinuation as a flanking measure of oncological therapy, which should be further investigated in the future.

## Figures and Tables

**Table 1:** Patient characteristics.

All Patients with rHCC after LT	*n* = 112	
Sex (%)		
male	100 (89.3)	
female	12 (10.7)	
Etiologies of HCC (%)		Recurrence after LT
HCV	42 (37.5)	33 (78.6)
HBV	15 (13.4)	6 (40)
alcohol	39 (34.8)	2 (0.05)
cryptic	11 (9.8)	-
hereditary disorders	3 (2.7)	-
autoimmune	2 (1.8)	-
Comorbidities (%)		
Diabetes mellitus	33 (29.5)	
Obesity (BMI > 30 kg/m^2^)	17 (15.2)	
Arteriosclerosis	10 (8.9)	
COPD	7 (6.3)	
Edmonson–Steiner Grade of HCC (%)		
G1	5 (4.8)	
G2	67 (64.4)	
G3	32 (30.8)	
Re-transplantation (%)	3 (2.7)	
Combined kidney transplantation (%)	3 (2.7)	
Median age at LT in years (min–max; Q1–Q3)	58 (31–72; 53–62)	
Date of LT (%)		
1989–1999	31 (27.7)	
2000–2009	66 (58.9)	
2009–2019	15 (13.4)	
Within MILAN-criteria according to histopathology (%)		
yes	28 (25%)	
no	75 (76%)	
Onset of rHCC (*n* = 108) (%)		
<2 years	68 (63.0)	
>2 years	40 (37.0)	
Median time to rHCC in months (min–max; Q1–Q3)	16.0 (1.0–230.0; 8.3–44.0)	
Median time of survival after rHCC in months (min–max; Q1–Q3)	10.6 (0.3–228.7; 3.3–22.9)	
Median AFP-levels in ng/mL (min–max; Q1–Q3)		
before LT (*n* = 96)	45.5 (1.0–1,072,817.0; 8.0–355.8)	
before rHCC (*n* = 78)	7.5 (1.0–124,254.0; 3.0–98.8)	
at rHCC (*n* = 75)	72.0 (1.0–605,505.0; 5.0–954.0)	
rHCC manifestation at time of diagnosis (%)		
liver only	15 (13.4)	
extrahepatic	56 (50)	
combined	32 (28.6)	
Oncological regimen for rHCC (*n* =103)		
Curative (%)	59 (57.3)	
Palliative (%)	44 (42.3)	
IS regimen (*n* = 103)	before rHCC	after rHCC
CNI-mono (%)	34 (33.0)	28 (27.2)
mTORI-mono (%)	7 (6.8)	18 (17.5)
CNI + MMF (%)	35 (34.0)	22 (21.3)
CNI + GC (%)	9 (8.7)	7 (6.8)
CNI + mTORI (%)	9 (8.7)	11 (10.7)
Others (%)	9 (8.7)	14 (13.6)
no IS (%)	0 (0)	3 (2.9)
Status at last follow-up (*n* = 112)		
alive (%)	9 (8.0)	
deceased (%)	103 (92.0)	
tumor progression	107 (95.5)	
others	5 (4.5)	

rHCC—recurrent hepatocellular carcinoma; LT—liver transplantation; HCV—hepatitis C virus; HBV—hepatitis B virus; AFP—alpha-fetoprotein; CNI—calcineurin inhibitor; mTORI—mTOR inhibitor; MMF—mycophenolate mofetil; GC—glucocorticoid; IS—immunosuppression; IQR—interquartile range; COPD—chronic obstructive pulmonary disease; G1—abundant cytoplasm; minimal nuclear irregularity; G2—prominent nucleoli, greater irregularity; G3—increased nuclear polymorphism, angulation of nucleoli, tumor giant cells; G4—poorly differentiated, marked nuclear polymorphism, hyperchromatism, anaplasia.

**Table 2:** Multivariate analysis for impact on survival after rHCC after liver transplantation.

Parameters	*n*	*p*	Hazard Ratio	95% CI	
				Lower	Upper
Age	90	0.35	1.0		
Obesity	17	0.40	0.74	0.37	1.50
Diabetes mellitus	30		0.95	0.56	1.59
Oncological therapy					
surgery	37	<0.000	0.35	0.20	0.61
radiotherapy	15	0.131	0.57	0.27	1.18
palliative (reference)	38	0.001			
Recurrent HCV-infection					
Yes	26	0.93	1.03	0.54	1.95
No	7	0.7	0.82	0.30	2.23
Without HCV at LT (reference)	57	0.91			
Recurrent HBV-infection					
Yes	5	0.31	1.68	0.61	4.62
No	8	0.56	0.76	0.30	1.94
Without HBV at LT (reference)	77	0.46			
Histological grading					
G1	2	0.243	0.41	0.09	1.84
G2	59	0.007	0.46	0.29	0.82
G3 (reference)	29	0.025			
Extent of recurrence		0.007	0.50	0.30	0.83
Single-organ	44				
Multi-organ	46				
Restrictive immunosuppression	59	0.026	0.55	0.32	0.93

rHCC—recurrent hepatocellular carcinoma; CI—confidence interval; LT—liver transplantation.

## Data Availability

The data presented in this study are available on request from the corresponding author. The data are not publicly available due to conditions of the ethics committee of our university.

## Abbreviations

AFP	alpha-fetoprotein
BSC	best supportive care
CCC	cholangiocellular carcinoma
CNI	calcineurin inhibitors
CYA	cyclosporine A
HBV	hepatitis B virus
HCV	hepatitis C virus
HCC	hepatocellular carcinoma
IQR	interquartile range
LT	liver transplantation
MMF	mycophenolate mofetil
MOA	multi-organ affection
MWA	microwave ablation
mTORI	mammalian target of rapamycin inhibitors
NA	nucleos(t)id-analog
PCR	polymerase chain reaction
RFA	radio frequency ablation
RFS	recurrence free survival
rHCC	recurrent hepatocellular carcinoma
RIM	restrictive immunosuppressive management
SOA	single-organ affection
TACE	transarterial chemoembolization
